# 1653. Cost effectiveness of Levofloxacin Vs Amoxicillin/Clavulanate And Ciprofloxacin In The Outpatient Management Of Low-Risk Febrile Neutropaenia In Children With Cancer: A Randomised Control Study.

**DOI:** 10.1093/ofid/ofad500.1487

**Published:** 2023-11-27

**Authors:** Reham Khedr, Ebtihal Abdelaziz, hadir Al mahellawy, Nashwa Ezz El-Deen

**Affiliations:** Children's cancer hospital Egypt 57357/ National Cancer Institute - Cairo university, cairo, Al Qahirah, Egypt; National Cancer Institute - Cairo university, cairo, Al Qahirah, Egypt; National Cancer Institute - Cairo university, cairo, Al Qahirah, Egypt; Children cancer hospital Egypt 57357/ National Cancer institute - cairo university, Cairo, Al Qahirah, Egypt

## Abstract

**Background:**

outpatient management of low-risk fever and neutropenia should be implemented if close monitoring is accessible and patient compliance is feasible.In this study, we aimed to assess the efficacy and safety of single-agent Levofloxacin versus the Augmentin /ciprofloxacin regimen used in our institute.

**Methods:**

This is a randomized prospective interventional 2 arm study of low-risk febrile neutropenia patients presenting to the emergency department at the National Cancer Institute, Cairo University starting from December 2021 to July 2022. Eligible patients were children< 18 years with Low-risk febrile neutropenia without an aetiological diagnosis.Patients were randomized to double-agent ciprofloxacin and amoxicillin-clavulanic acid in comparison to single-agent levofloxacin. Follow up of the outpatient cases on; Day 1, Day 3, and Day 7 for Resolution of infection and stopping of antibiotics regardless of neutropenia. Drug-related side effects encountered in both arms of the study were reported. A decision analytic model was created to compare the two different treatment strategies Outcome measures were quality-adjusted FN episodes, costs, and incremental cost-effectiveness ratios (ICER). The clinical parameters of the model were derived mainly from data collected at National Cancer Institute. Health effects were expressed in terms of QALY gain.One-way sensitivity analysis (DSA) was conducted.

**Results:**

200 patients (100 in each group) were enrolled. 100% of patients achieved marrow recovery in both arms by D7. Fever subsided in 100% on the Levofloxacin arm compared to 60% in the other group. only 1 patient on the double agent arm was upgraded to HR.Levofloxacin was tolerable in all patients with no significant side effects, while patients in the double agent arm; 4 had diarrhea, 2 nausea, and 2 complained of a bad drug taste. Levofloxacin is the dominant strategy versus Augmentin/ciprofloxacin with incremental QALY 0.0001and lower cost 62.4996 L.E in the treatment of home-based care of febrile neutropenia with a willingness to pay threshold of 77,520 LE per QALY (1 GDP/CAPITA)

**Figure:**
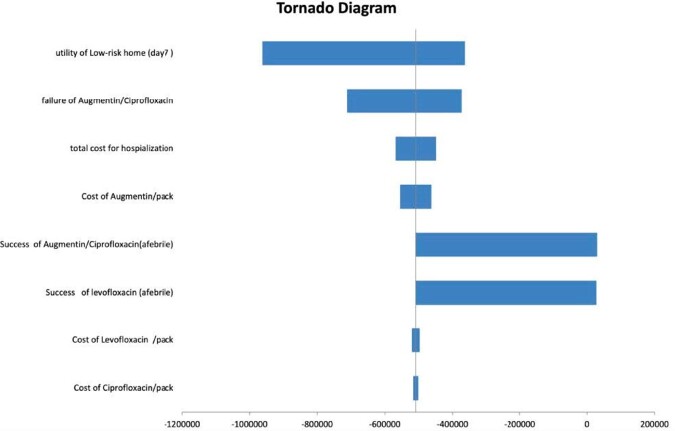

Figure 1

**Conclusion:**

Levofloxacin is cost effective and can be administered safely in children with low-risk FN, however close follow-up for long-term side effects and emerging bacterial resistance is warranted.

**Disclosures:**

**All Authors**: No reported disclosures

